# Organosilica-Based Membranes in Gas and Liquid-Phase Separation

**DOI:** 10.3390/membranes9090107

**Published:** 2019-08-22

**Authors:** Xiuxiu Ren, Toshinori Tsuru

**Affiliations:** 1 Jiangsu Key Laboratory of Fine Petrochemical Engineering, School of Petrochemical Engineering, Changzhou University, Changzhou 213164, China; 2 Separation Engineering Laboratory, Department of Chemical Engineering, Hiroshima University, 1-4-1 Kagamiyama, Higashi-Hiroshima 739-8527, Japan

**Keywords:** organosilica, hybrid membrane, hydrothermal stability, gas separation, liquid separation

## Abstract

Organosilica membranes are a type of novel materials derived from organoalkoxysilane precursors. These membranes have tunable networks, functional properties and excellent hydrothermal stability that allow them to maintain high levels of separation performance for extend periods of time in either a gas-phase with steam or a liquid-phase under high temperature. These attributes make them outperform pure silica membranes. In this review, types of precursors, preparation method, and synthesis factors for the construction of organosilica membranes are covered. The effects that these factors exert on characteristics and performance of these membranes are also discussed. The incorporation of metals, alkoxysilanes, or other functional materials into organosilica membranes is an effective and simple way to improve their hydrothermal stability and achieve preferable chemical properties. These hybrid organosilica membranes have demonstrated effective performance in gas and liquid-phase separation.

## 1. Introduction

Molecular separation and purification are very important processes in chemical engineering and environmental protection. Distillation, adsorption, absorption, extraction and membrane separation are the current technologies that are commonly used for separation. Among them, membranes-based technology has been increasingly attractive applied in gases and liquids-phase separation due to energy-savings, cost-effective and environmental-friendly operations [1]. Till now, thousands of membranes have been synthesized and evaluated. As one important type of inorganic membranes, silica-based membranes have molecular pore sizes and high thermal stability with excellent separation performance. Most silica membranes prepared using tetraethoxysilane (TEOS) show pore sizes in the range of 0.3–0.4 nm, which are particularly suited to the separation of smaller gases from other gases with relatively large diameters [2,3,4]. In the hydrogen separation, silica membranes generally show H_2_ permeances ranging from 10^−6^–10^−8^ mol·m^−2^ s^−1^ Pa^−1^) with H_2_/N_2_, H_2_/CH_4_, and H_2_/CO_2_ selectivities over hundreds to thousands, which are sufficient for the demands of industrial separations [5,6]. 

However, pure silica membranes are known for their low level of hydrothermal stability wherein water molecules can degrade the network of silica by hydrolysis of Si–O–Si even at low temperature, resulting in a loss of separation ability [7]. In addition, small pore sizes of pure silica membranes increase unnecessary transport resistance in the separation of H_2_ from larger molecules such as separation systems for H_2_/C_3_H_8_ and H_2_/SF_6_, etc. These disadvantages restrict the membranes to most gas or liquid-phase applications. After 1998, many scientists attempted to find methods to modify silica membranes, and these efforts are documented in several reviews [8,9,10,11,12,13].

In these silica-based membranes with SiO_2_ as a structure, a new type of membranes categorized as silsesquioxane (RSiO_1.5_) derived from organoalkoxysilanes was developed in 2008, to enhance hydrothermal stability and allow tuning of the network structures that could overcome the drawbacks of pure silica membranes [14]. These types of membranes usually contain various organic functional groups in the inorganic Si–O–Si networks, which were ever given names such as organic-inorganic silica membrane, hybrid silica membrane, silica-based membrane, organosilica membrane or hybrid organosilica membrane. Actually, too many names to describe these new type membranes are confused for other researchers. In the past few years, the name “organosilica” seems favored to represent these membranes. In addition, a one-word name is easier to remember and describe. We believe the term “organosilica” is suitable and used in the following discussion. The name “hybrid organosilica” is used to describe organosilica membranes that are doped with other materials.

**Figure 1:**
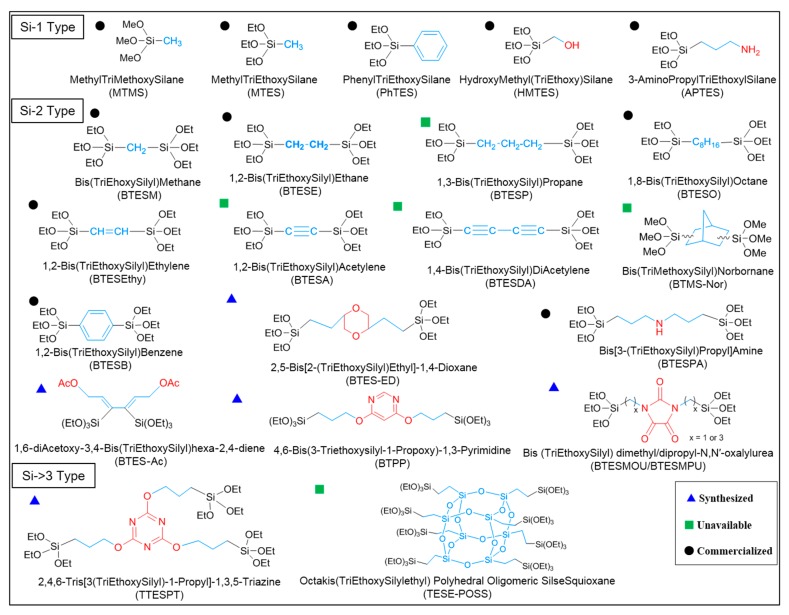
Most of the organoalkoxysilane materials along with their chemical structures. (Et: ethyl; Me: methyl; Letters in brackets are their abbreviation name.).

**Figure 2:**
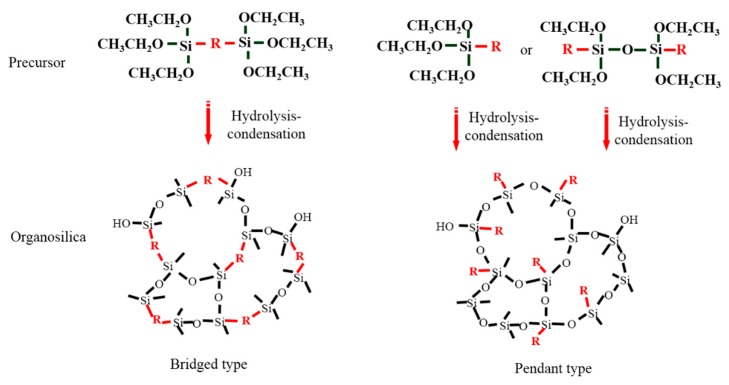
The represented bridge-type and pendant-type precursors and their organosilica structures formed (R: organic groups).

Till now, dozens of organoalkoxysilane materials have been proposed as precursors to prepare organosilica membranes, and most of precursors are shown in Figure 1. These materials can be divided into Si-1, Si-2 and Si-3 dependent on the number of silicon atoms in one molecule. The alkoxy groups in the precursor are usually ethoxy –OCH_2_CH_3_, and in a few cases, methoxy –OCH_3_. The ethyl or methyl in alkoxy groups will be lost in the formation of networks. The represented precursor types and their organosilica structures formed are shown in Figure 2. Based on the networks, bridged-type and pendant-type are more commonly used to classify these organosilica membranes. The bridged-type consists of organic groups between two silicon atoms as O–Si–R–Si–O, and pendant-type is terminal organic groups bonding with silicon atoms as R–Si–O–Si–(R’ or O). Three of the alkoxy groups are commonly connected with one Si atom, leading to a formation network of SiCO_1.5_, which is often referred to silsesquioxane. The organic groups R can be more than one connected with Si atoms in the networks.

The existence of organic groups in the organosilica networks effectively decreases the content of Si–OH and repels water attaching silicon-oxygen bond. This property reduces the decomposition of Si–O–Si networks and enhances hydrothermal stability in the presence of water vapor under high temperature. It is important to note that pore sizes and surface properties including affinity with certain molecules such as hydrophilicity or hydrophobicity can be controlled by these organic groups. For example, groups of –CH_2_–, –CH_2_–CH_2_–, –CH_2_–CH_2_–CH_2_–, –(CH_2_)_6_–, –(CH_2_)_8_–, and phenyl as bridged types have been reported to tune the pore size of membranes [15,16,17]. These types of organosilica membranes with thermally and chemically stable structures showed good performance in gas separation, pervaporation and reverse osmosis (RO) applications. In particular, 1, 2-bis(triethoxysilyl)ethane (BTESE) has a bridging unit consisting of ethylidene between two Si atoms (≡Si–CH_2_–CH_2_-Si≡) and is used extensively to prepare organosilica membranes with controlled pore sizes. The progress in the development of BTESE membranes for gas and liquid separation has been phenomenal in recent years, and some of which have been applied in industries [18]. Organic groups with the same carbon number but different alkyl types such as –CH_2_–CH_2_–, –CH=CH–, –C≡C– have also been investigated. These showed different surface affinity with water and produced good performance in desalination application [19,20]. A pendant-type membrane containing Si–F bonds, which was fabricated using triethoxyfluorosilane (TEFS), showed improved hydrothermal stability and enhanced affinity with hydrocarbons, compared with TEOS-derived silica membranes [21]. Another pendant-type organosilica membranes with primary, secondary or tertiary amine groups are used to enhance affinity for CO_2_ separation [22,23]. 

In general, organic materials have low thermal stability, and therefore easily decompose at temperature above 100 °C. However, the organic groups in the silica network can be kept stable at 300 °C even under an oxidative atmosphere, which is attributed to the protection imparted by the connected inorganic Si–O–Si structure [24,25,26]. Thus, organosilica membranes possess a higher level of thermal stability than organic membranes, and also have shown hydrothermal stability compared with pure silica membranes, which can expand the scope of applications and increase the probability of utility in industry. The preparation of organosilica membranes and their applications are summarized in Section 2.

Pure organosilica membranes exhibit interesting performance in the separation of H_2_ from large molecules such as CH_4_ or C_3_H_8_, but lack the separation performance required to separate H_2_ from CO_2_, CO or N_2_ due to the loose structures and low affinity to H_2_. For some special applications, such as H_2_/CH_4_, H_2_/CO or H_2_/CO_2_ in the water-gas shift (WGS) or steam-methane reforming (SMR), purification of H_2_ at a high temperature in the presence of water vapor is required. Thus, high separation performance and high hydrothermal stability are the two important directions for applications in WGS and SMR. Using functional materials to modify organosilica membranes is an effective and simple way to increase membrane performance during preparation. Thus, hybrid organosilica membranes are increasingly being developed via incorporation of metals, inorganic ions, polyhedral oligomeric silsesquioxane, even polymer and other alkoxysilanes into organosilica sols with desired properties [27,28,29,30,31,32,33]. In particular, two or three organoalkoxysilane materials in a complex mixture have also been studied. A hydrophilic hybrid membrane designed by incorporation of hydroxymethyl(triethoxy)silane (HMTES) with –OH groups into BTESE obtained a water permeance that was about 4 times higher than that of pure BTESE membrane in reverse osmosis application [32]. Hybrid organosilica membranes by functional materials doping and their applications are summarized in Section 3.

## 2. Pure Organosilica Membranes

Organosilica membranes are usually prepared on ceramic supports. As the supports are mostly macroporous, an intermediate layer is pre-coated onto supports to form mesopores, and then the organosilica sols/precursors are then coated/deposited on the intermediate layer to form a thin separation layer. This method can prevent small molecular organosilica sols/precursors from penetrating support and forming a thick or defect separation layer. The intermediate layer is usually hydrophilic to support -OH groups which can form covalent bonds with organosilica layers. In recent years, hydrophobic intermediate layer, interlayer free, or layered coating on polymer substrates to prepare organosilica membranes have been investigated in effects to avoid water condensation for gas transport, reduce the transport resistance or save cost for industrial applications [4]. No matter how the layer is changed, the basic principle is preventing the penetration of separation materials into the support and obtaining an intact and thin layer for separation. 

**Figure 3:**
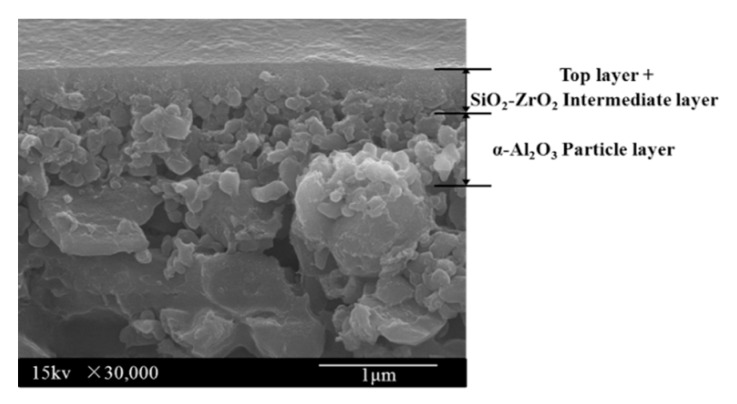
A cross-sectional SEM image on the constructions of BTESE membranes including intermediate and support layer. Reproduced from [16].

Organosilica separation layer is usually prepared by sol-gel (SG) method and chemical vapor deposition (CVD) method. The SG method is more commonly used to produce layers that are very thin and preferable for gas transport. A scanning electron microscope (SEM) image of the cross-section of a BTESE membrane prepared by this method is shown in Figure 3 [16]. It can be observed that the thickness of top layer (BTESE separation layer) is less than 500 nm including intermediate layer, which is much thinner than most zeolite membranes. The network structures and chemical properties are not only dependent on the organic groups, but also are greatly affected by the synthesis conditions, which are key factors in the process of membrane preparation. The performance of these organosilica membranes prepared by different conditions and precursors in the gas and liquid-phase separation are compared in this section. 

### 2.1. Sol-Gel Method

The term “sol−gel” was first invited to describe silica sols by Graham in 1864, and a detailed history and publications on the development of sol-gel methods in preparation of silica-based materials have been reviewed [34]. It is one of the most feasible and cost-effective methods to form an integrated network through hydrolysis (sol) and poly-condensation (gel) of alkoxysilanes. The principle is shown in the reaction (1)–(3). The alkoxysilanes are hydrolyzed with water to form Si–OH, and condensed to form Si–O–Si networks.

≡Si–OEt + H_2_O ⇔ ≡Si–OH + EtOH (1)

≡Si–OH + ≡Si–OH ⇔ ≡Si–O–Si≡ + H_2_O (2)

≡Si–OEt + ≡Si–OH ⇔ ≡Si–O–Si≡ + EtOH
(3)

**Figure 4:**
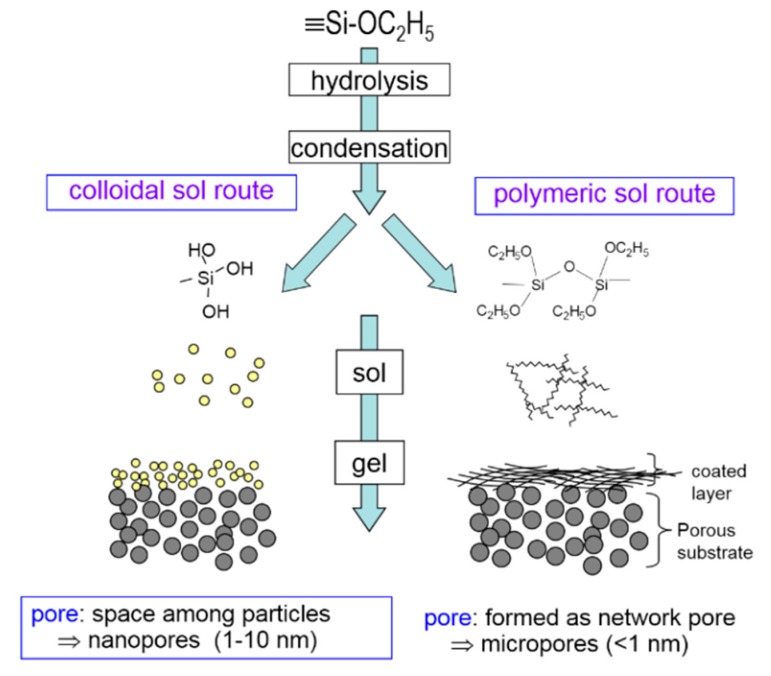
The colloidal and polymeric route in sol-gel process. Reproduced from [10].

Two types of sols can be prepared via the colloidal route and polymeric route, depending on the reaction rate. The schematic process is shown in Figure 4. The relative hydrolysis and condensation rates are very sensitive to the catalyst and media. Using a base as the catalyst and excessive water creates reaction rates that are very fast, and colloidal sols are generally formed. The membranes prepared by colloidal sols are usually mesoporous, which make them suitable for nanofiltration, ultrafiltration or use as intermediate layers. On the contrary, by using an acid as the catalyst with excessive alcohol solvent, the reaction rate is slow with a partial hydrolysis. This results in a linear polymeric sol with micropores that can be used for molecular sieving separation. When sols are prepared, steps of coating sols on a support, gel formation, drying and thermal treatment are followed to prepare organosilica membranes. Therefore, the influencing factors such as synthesis composition of sols and the calcination temperature for gels in the formation of networks and other factors are summarized in Table 1 and discussed in a later section. 

#### 2.1.1. Effect of the Composition Ratio 

In the preparation of sols, the precursor generally is dissolved in a solvent first, then mixed with water and a catalyst. The water molar ratio of H_2_O/Si (WR) and acid catalyst molar ratio of H^+^/ Si (AR) as important factors have been examined in different ways. 

Molar ratios of H_2_O/BTESE (WR = 6–240) have been reported in the preparation of BTESE membranes via sol-gel method [35]. A higher WR increased the hydrolysis degree of ethoxides in the precursor, and produced a denser network. The selectivity of H_2_/methylcyclohexane was increased from 100 to 10,000 by increasing WR from 6 to 240. A similar result was also found in the preparation of membranes using triethoxysilane (TRIES) as a precursor [36]. The TRIES-derived networks showed a decreased pore size with an increase in the WR, which was ascribed to a higher degree of hydrolysis and polymerization of ethoxy groups (–OEt) with high content of water.

**Figure 5:**
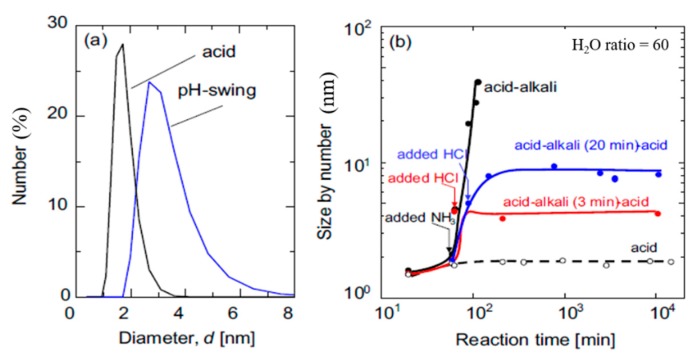
(**a**) Dynamic light scattering for pH-swing sol and acid sols. (**b**) Time course of BTESE-derived sols in acid, acid-alkali and acid-alkali-acid. Reproduced from [39].

The effect of acid molar ratio (AR = 0.01 and 0.1) on membrane performance under a low water ratio of 6 was reported by Castricum’s group [37]. BTESE membranes prepared under AR = 0.01 condition showed much higher H_2_/N_2_ selectivities (50–80) compared with that prepared under AR = 0.1. With more acid, compressive forces generated in the drying process of sols are balanced by the positive charge, resulting in large pores that decreased the separation ability. The pore formation mechanism for AR in the range of 10^−4^–10^0^ was evaluated by Tsuru’s group at high water ratios of 60–240 [38]. It was found that the hydrolysis rate was faster at a higher acid concentration, leading to a larger size and higher density of silanols in the sols. In the drying process, the condensation rate of silanols was increased first but then it was decreased as the AR increased. Therefore, the BTESE membrane prepared with AR = 10^−2^ showed the highest permselectivity for H_2_/N_2_ of 40–70 and H_2_/CF_4_ of around 20,000 with a H_2_ permeance of 16 × 10^−7^ mol m^−2^ s^−1^ Pa^−1^ at 200 °C. It was explained by the existence of sufficient numbers of silanol groups that were completely condensed during firing and resulted in small pores suitable of H_2_ transport. By Normalized Knudsen-based Permeance (NKP) method to evaluate the pore size of membranes, it was confirmed that membranes prepared at a lower AR = 10^−2^ (0.44 nm) had a pore size that was smaller the than that with a higher AR = 10^0^ (0.54 nm) [38]. In the pervaporation of alcohols, the membrane prepared at AR = 10^−2^ also showed a larger separation factor due to smaller pores. The water affinity with membranes measured by contact angle was found not to be affected by the acid concentration. As the sols and ultimate network formed by thermal treatment were sensitive to the acid or base catalyst, a novel process by pH-swing method was reported to obtain sols with a larger size but with a similar pore size network compared with those synthesized only under acid conditions [39]. In the procedure of sol preparation, the solution was treated in the acidic-alkali-acidic three steps, as shown in Figure 5. The pH was tuned to 2 via the use of acid and changed to pH = 10 using alkali for a very short period to accelerate the growth of the sol size. The pore size of membranes was not changed by pH-swing method compared with acid method, but sols with larger particle sizes showed less penetration into the intermediate layer. Thus, the permeance of gas was improved without sacrificing separation selectivity. 

In the synthesis process, a media is generally needed to disperse the reagent and generate homogenous sols. As the organoalkoxysilane precursor is water-insoluble, ethanol as the solvent is commonly used as the media. However, research on the solvent effect has been scant. Actually, the solvent ratio has also been found to affect the networks with the exception of the acid and water ratios [40]. Under higher EtOH/BTESE ratios, smaller sol sizes and unimodal particle size distributions are preferably formed. The BTESE membranes prepared from these optimized sols exhibited a H_2_ permeance of 4.2 × 10^−7^ mol·m^−2^·s^−1^·Pa^−1^, and permselectivities for H_2_/CO_2_, H_2_/N_2_ and H_2_/CH_4_ were 9.5, 50 and 68, respectively.

#### 2.1.2. Effect of Calcination Temperature

After coating the organosilica sols onto the intermediate layer, drying and thermal treatment are followed to evaporate the solvent and further poly-condensation of gels. The thermal treatment is very important because the gels are connected with each other to form network structures with a defect-free layer at this stage. It is usually carried out by placing the support into a furnace at high temperature to be calcinated for a certain time. Thus, the effect of calcination temperature on the membrane structures and properties is significant. de Vos and Verweij reported the pure silica membranes calcinated at 400 and 600 °C [2]. The membrane prepared at 400 °C showed a very high H_2_ permeance of 20 × 10^−7^ mol m^−2^ s^−1^ Pa^−1^ with H_2_/CH_4_ selectivity above 500, while the membrane calcinated at 600 °C showed a lower H_2_ permeance but higher separation selectivity than that prepared at 400 °C. The higher calcination temperature led to a higher degree of condensation of terminal hydroxyl groups and formed a smaller pore size structure on the internal surface. A similar trend has also been reported on polyhedral oligomeric silsesquioxane (POSS)-derived silica membranes fired at 550 °C whereby the pore size distribution was shifted to a smaller size, in comparison with firing at 300 °C, due to sintering of the amorphous silica structure [41]. 

In the organosilica membranes, the calcination temperature on BTESE membranes for reverse osmosis (RO) desalination were reported [42]. The membranes calcinated at 300 °C exhibited a higher salt rejection and a lower water flux compared with those calcinated at 100 °C. A denser organosilica network structure was obtained at higher temperatures due to the acceleration of the condensation reaction of silanol groups (dehydroxylation) that resulted in a smaller pore size structure. Bis(triethoxysilyl)-methane (BTESM)-derived organosilica membranes that were fabricated at 200, 350 and 600 °C for separation of propylene/propane showed the same conclusion about the effect of temperature [43]. The membranes fired at 200 °C showed a higher permeance of C_3_H_6_ and a lower selectivity of C_3_H_6_/C_3_H_8_ than membranes fired at 350 °C due to a larger pore size. It was confirmed by Fourier transform infrared spectrometer (FT-IR) spectra that the peak of silanol groups disappeared with an increase in calcination temperatures from 200 to 600 °C, indicating an enhancement of the condensation reaction of silanol groups. These results show that a higher calcination temperature accelerates the condensation of silanol groups, which results in smaller pore sizes. The selectivity is improved under higher calcination temperatures but at a cost of lowering permeance in gas and flux in liquid separation. 

#### 2.1.3. Effect of Other Factors

In the process of membrane preparation, dip-coating or wipe-coating method is commonly used to transfer the organosilica sols onto the support surface, and then followed by drying and thermal treatment. The support usually used either an α-Al_2_O_3_ tube or a disk, which is hard with good mechanical property. Recently, the use of polymers as supports to prepare BTESE-derived membranes has been reported due to their low cost and suitability for large-scale industrial manufacturing [44]. In the sol-gel process, spin-coating is a facile way to complete BTESE deposition on porous polysulfone supports instead of dip- or wipe-coating. The concentration of BTESE sols and the number of spin-coating cycles both play important roles in the formation of a continuous and defect-free membrane. On the other hand, solvent evaporation rate in the drying process is another important factor that influences membrane quality and uniformity. Methanol, ethanol and 1-propanol were reported as solvents in the preparation of BTESE sols [45]. Only sols using 1-propanol have enabled the successful preparation of BTESE membranes on a polysulfone (PSF) support via the spin coating method. The slower evaporation rate of 1-propanol was identical as spin coating conditions that led to a more uniform BTESE layer on the PSF film.

Instead of calcination of gels by thermal treatment, a novel approach via photo-induced processing was developed to prepare polymer-supported organosilica membranes at room temperature [46,47]. The effect of UV irradiation on the network formation is important. The photo- acid-catalyzed hydrolysis of methoxy groups was completed within a very short period of UV irradiation less than 30 s. The bis(trimethoxysilyl)ethane (BTMES) membrane prepared by this method have displayed a water permeance of 3.0 × 10^−6^ mol m^−2^ s^−1^ Pa^−1^ with a separation factor of 99 in the pervaporation of a 90 wt% isopropanol aqueous solution at 40 °C.

In addition to these researches on the compositions, calcination temperatures, deposition and condensation methods, many other parameters’ effects on the membrane preparation remain unclear. For example, HCl, HNO_3_ and CH_3_COOH were ever used as a catalyst to prepare organosilica membranes by several groups, but the effect of these acid types was few compared [48]. The effect of solution reaction temperature in the synthesis of sols and calcination time on the network formation was neither discussed in detail. For example, the sol synthesis at 25 or 60 °C, and firing for 30 min or 3 h, have been reported, but the effect on membranes have neither been compared nor investigated. Thus, many parameters should be further investigated in order to understand the mechanisms and kinetics of sol−gel reactions. Through an effective control of these conditions, it is possible to tailor the structures and properties of organosilica membranes via sol-gel method.

### 2.2. CVD Method

Chemical vapor deposition (CVD) is a technique to deposit layers on a substrate via the reactions of one or several gas phase precursors. Generally, a mixture of reactive compounds delivered by carrier gases (such as H_2_, N_2_, Ar) is flowed over the substrate in a vapor or gas phase and then the reaction occurs at a certain temperature. The pore structures are formed by the reactions of precursors, which can form pore sizes that are smaller than those membranes prepared via sol-gel method based on particle packing. Thus, the CVD membranes generally showed higher selectivities with lower level of gas permeance than those of SG membranes [49]. For example, phenyltriethoxysilane (PhTES) derived organosilica membrane via the CVD method showed a H_2_ permeance of 0.57 × 10^−7^ mol m^−2^ s^−1^ Pa^−1^ with H_2_/N_2_ selectivity of 33, while the PhTES membrane via the SG method showed a H_2_ permeance of 2 × 10^−7^ mol m^−2^ s^−1^ Pa^−1^ and H_2_/N_2_ selectivity of 7.4 with a large pore size of 0.76 nm [50,51].

Dimethoxydimethylsilane (DMDMS)-derived organosilica membrane was prepared via a counter-diffusion CVD method at high temperature using oxygen as a co-reagent deposition [49]. The pore size of DMDMS membrane was 0.35 nm, resulting in a very high ideal selectivity of H_2_/N_2_ higher than 2000 with H_2_ permeance of 2 × 10^−7^ mol m^−2^ s^−1^ Pa^−1^ after 60 min deposition. Another hybrid organosilica membrane that used 3-aminopropyltriethoxysilane (APTES) and TEOS as the co-precursors was also successfully prepared by one-side CVD method at 673 K reaction for 1–4 h [52]. The hybrid membrane showed a small pore size of 0.44 nm and reserved amino groups to facilitate CO_2_ transport, and achieved CO_2_/CH_4_ selectivity of 40. In recent year, by optimizing precursors with phenyl groups, organosilica-based membranes were prepared via a CVD method and also showed high H_2_ permeances (more than 10^−6^ mol m^−2^ s^−1^ Pa^−1^) and H_2_/SF_6_ selectivity by shifting their pore size from 0.3 to about 0.5 nm. The relatively large pore sizes of CVD membranes are similar to SG membranes that have pore sizes of 0.4–0.6 nm (BTESE), which will have potential applications in hydrogen-organic gas separation [53,54].

Organosilica membranes prepared via the thermal CVD method have good gas performance, but the types of materials are limited due to the requirement of temperatures above 500 K. The organic groups are probably decomposed particularly under O_2_ atmosphere, and cause a loss of functional properties. Plasma technique can form highly cross-linked films at relatively low temperatures or even at room temperature. Thus, a plasma-enhanced CVD (PECVD) method has been extensively applied for membrane production [55]. In addition, the pressure at either low pressure or atmospheric pressure are used in PECVD [56,57]. Several pendant-type precursors such as hexamethyldisiloxane (HMDS), trimethylmethoxysilane (TMMS), and methyltrimethoxysilane (MTMS) successfully were used to fabricate membranes via PECVD at room temperature under vacuum pressure [58,59]. A deposition time of less than 5 min by plasma technique can form a dense layer on supports for gas separation. The plasma working gases of pure Ar, Ar/O_2_ and Ar/N_2_ mixtures were investigated under atmospheric pressure [60]. The Hexamethyldisiloxane derived membrane using Ar/N_2_ plasma displayed highly efficient gas separation with He/N_2_ selectivity of 196. These results reveal that PECVD is an attractive technique to fabricate organosilica membranes at low temperature, which probably helps to use inexpensive polymers as supports.

### 2.3. The Effect of Organic Groups 

Bridged- and pendant-types of organosilica membranes can be prepared using a similar method. Both types can be used to tune pore size and design properties of membranes via SG or CVD method, but the two types of membranes display different characteristics and performance in gas and liquid-phase separation. The bridged-organosilica membranes have been more extensively investigated and generally show higher performance than pendant membranes. On the other hand, the surface properties that include hydrophilicity-hydrophobicity and affinity are more easily controlled by the pendant groups. Therefore, a better understanding of the properties of hybrid silica membranes prepared using different types of alkoxysilanes and their performance becomes very important in providing directions for the development of high-performance membranes with efficient applications. In addition, the synthesized factors and other transport resistance (such as thickness) should also be considered for the different separation performance. For example, extensive reports on BTESE membranes have shown differences in H_2_ permeance that ranged from 10^−5^–10^−7^ mol m^−2^ s^−1^ Pa^−1^ [15,16,26,37]. The comparison of bridged-organosilica and pendant-organosilica membranes are summarized in Table 2, Table 3, Table 4 and Table 5 in gas and liquid-phase separations, respectively.

In Table 2, nearly all the bridged organosilica membranes showed H_2_ permeance in the range of 10^−5^–10^−7^ mol m^−2^ s^−^^1^ Pa^−^^1^, which are higher than pure silica membranes, and also higher than most polymers and some zeolite membranes [6,75,76]. The permselectivity for H_2_/N_2_ was moderate due to a loose network above 0.4 nm, but in the separation of H_2_ from large molecules such as C_3_H_8_, SF_6_ etc., the membranes showed very large selectivities over thousands [39]. In a similar manner, as shown in Table 3, for pervaporation of alcohol/water systems, the bridged organosilica membranes exhibited a high level of water flux and permeated water concentration than polymers. Compared with zeolite membranes, organosilica membranes showed a similar water flux with lower separation factor [77]. However, NaA-type zeolite membranes could not be operated in solutions with a high water content due to low stability under high temperatures in pervaporation [78]. Instead, in either a 50 wt% isopropanol aqueous solution at 75 °C or in the vapor permeation of a high-water-content stream at 100 °C, the organosilica membranes showed an excellent stability for long-term pervaporation with a water permeation flux of 6 kg m^−^^2^ h^−^^1^ and separation factor of 125 [68]. In the desalination applications, these organosilica membranes exhibited excellent chlorine-resistance stability [79]. Even the membranes were under chlorine concentrations that reached 35,000 ppm h, there were no obvious change in the separation performance, which was ascribed to a main structure consisting of chemically strong bonds such as Si–C and Si–O, and lacked of amide linkages which are sensitive to attack by aqueous chlorine.

**Figure 6:**
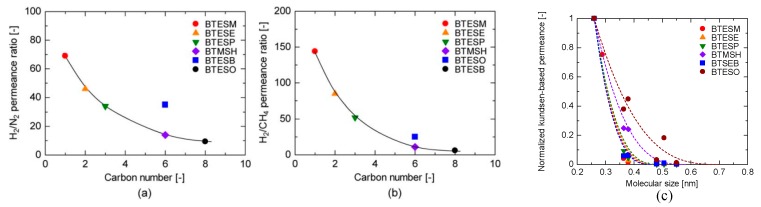
The permeance ratios of (**a**) H_2_/N_2_ and (**b**) H_2_/CH_4_ at 200 °C; (**c**) pore size prediction based on a NKP method as a function of the number of carbon linking units. Reproduced from [15] in context and supporting information.

**Figure 7:**
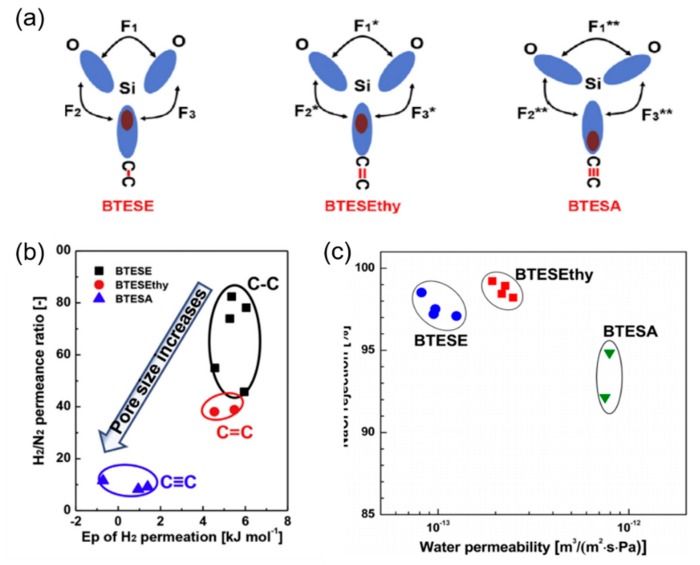
(**a**) Bonding structure model, (**b**) H_2_/N_2_ permeance ratio at 200 °C and (**c**) water permeability versus salt rejection (25 °C, 1.15 MPa, and 2000 ppm of NaCl) for BTESE, BTESEthy, and BTESA membranes. Reproduced from [19,80].

In these bridged organosilica membranes, carbon number and their valence bond between two Si atoms were very interestingly investigated. As the carbon number increased, the selectivity of H_2_/N_2_ and H_2_/CH_4_ was decreased, as shown in Figure 6. It can be found that the network pore sizes were successfully controlled by using different carbon number (Figure 6c). Single C–C, double C=C or triple C≡C bonds in the precursors were used to prepare organosilica membranes referred to as BTESE, BTESEthy and BTESA, respectively. The O–Si–O bond angle was greatly affected by these bonds, as shown in Figure 7. The pore size and water contact angle were also different for these membranes. The pore sizes were 0.42, 0.43, and 0.52 nm for BTESE, BTESEthy and BTESA, respectively, which resulted in a decrease selectivity of H_2_/N_2_ [80]. Water contact angles were 66°, 50°, and 48° for BTESE, BTESEthy and BTESA, and the BTESA membranes showed a higher water permeability in desalination [19].

In Table 4 and Table 5, the pendant-types organosilica membranes show a relatively lower performance in gas and liquid-phase separation than that bridged-types. But pendant organic groups, such as –NH or –OH, can be designed to facilitate CO_2_ or water transport, and more functional groups can be easily obtained from commercialization, by comparison with bridged types.

## 3. Hybrid Organosilica Membrane 

In the process of membrane preparation, organosilica sols or organoalkoxysilane precursors doped with functional materials are recognized as hybrid organosilica membranes. A desired pore size and affinity with molecules as well as hydrothermal stability, can be expected via the hybrid route by the appropriate selection of material and composition. In this section, metal, alkoxysilane, and other material dopants are summarized in detail and compared with undoped organosilica membranes. 

### 3.1. Metal Doping

Till now, Pd, Zr, Cr, Co, Al, Ag, Nb, Ta, La and Y have been incorporated into organosilica sols to prepare a hybrid organosilica membrane [27,81,82,83,84,85]. Before the organosilica membranes were extensively investigated, metals such as Zr, Ti, Pd and Co were introduced into pure silica membranes derived by TEOS to enhance the affinity with gas or water and improve hydrothermal stability [4,86,87]. Kanezashi et al. doped 25–90 mol% Pd in the pure silica membrane, and obtained very high H_2_ permeances of 10–21 × 10^−7^ mol m^−^^2^ s^−^^1^ Pa^−^^1^ with H_2_/N_2_ permselectivity above 100 [88]. With 20 mol% Mg doping, Mg–SiO_2_ membranes obtained permselectivity for H_2_/CO_2_ more than 350 with a H_2_ permeance of 0.7 × 10^−7^ mol m^−^^2^ s^−^^1^ Pa^−^^1^ [89]. Among these metals, zirconium alkoxide has been extensively studied to prepare silica-zirconia membranes, due to strong affinity with water and good hydrothermal stability induced by Zr. Our group prepared a SiO_2_–ZrO_2_ membrane from silica-zirconia composite colloidal sols (molar ratio Si/Zr = 9/1), which showed pure water permeability ranging from 150–1500 × 10^−^^13^ m^3^ m^−^^2^ s^−^^1^ Pa^−^^1^ in the nanofiltration with controlled pore sizes from 1.0 to 2.9 nm [13]. This SiO_2_–ZrO_2_ membrane showed good stability in water for 100 days at 25 °C. Treatment in water at 90 °C for 4 h, the membrane was not damaged [90]. Instead, the water permeability was increased dramatically and stable for as long as 100 h in water. 

Based on these results we know that the silica membranes doped with metal ions showed greatly improved gas and liquid-phase separation performance as well as good stability in water. Organosilica membranes are more hydrothermally stable than pure silica membranes, and when doped with metal, will show a greater potential for industrial applications with a long lifetime use. For WGS or RSW reactions in particular, separations of H_2_/CO_2_ and H_2_/N_2_ are under humid process and high temperature, which requires the membranes with high level of hydrothermal stability. 

Metal salts and oxide are commonly used in the preparation of hybrid metal-doped organosilica membranes. Ten Hove et al. investigated the effect of zirconia concentrations when doped into BTESE-derived silica membranes (molar ratio Si/Zr = 1/0.1), and found that 0.2 mol/L silica-zirconia membrane showed permselectivities for H_2_/CO_2_, H_2_/N_2_ and H_2_/CH_4_ that were improved from 4 to 16, 12 to 100, and 12 to 400, respectively, compared with pure BTESE membranes [82]. A larger content of Zr in the BTESE membranes (Si/Zr = 1/0.34) reported by Qi’s groups exhibited a very high selectivity of H_2_/CH_4_ over 1150 when tested at 200 °C [91]. The metals of Nb and Pd were investigated by the same groups, and the 17%–33% Nb-BTESE membranes showed an excellent H_2_/CO_2_ permselectivity of 1500 with a relatively low H_2_ permeance of 0.2 × 10^−^^7^ mol m^−^^2^ s^−^^1^ Pa^−^^1^ [81,83,92]. The much high selectivity was due to smaller pores formed by metal-doping. The membranes maintained good stability in steam at 200 °C for 300 h. An opposite result of the pore size was observed in the preparation of 20% B-BTESE, 20% Ta-BTESE and 20% Nb-BTESE membranes that formed large open pores by doping metals [84]. These membranes exhibited higher H_2_ permeance but lower H_2_/CO_2_ selectivity than pure BTESE membranes. The reason remains unclear. 

**Figure 8:**
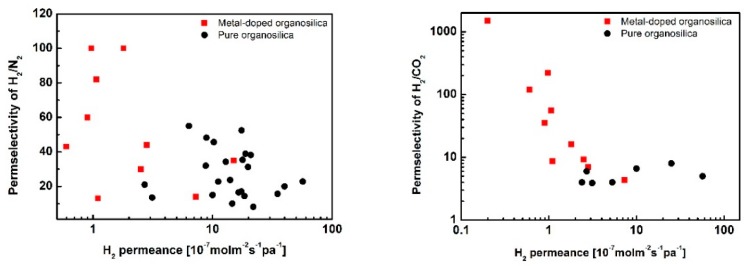
H_2_ separation performance of Metal-doped organosilica membranes and compared with organosilica membranes at 200 °C. Square data from Ref. [27,81,82,83,92,93], cycle data from Ref. [16,27,35,94].

Gas separation performance for metal-doped organosilica membranes is shown in Figure 8. It can be observed that metal-doped BTESE membranes showed H_2_ permeance ranging from 10^−^^6^–10^−^^8^ mol m^−^^2^ s^−^^1^ Pa^−^^1^, and the permselectivities for H_2_/N_2_ and H_2_/CO_2_ that ranged from 10 to 1500. Pure organosilica membranes showed higher permeance in the range of 10^−^^5^–10^−^^7^ mol m^−^^2^ s^−^^1^ Pa^−^^1^, with the permselectivities for H_2_/N_2_ and H_2_/CO_2_ that ranged from 2–60. According to these reports, we can find that the doped metal reduces the pore size of organosilica membranes, which makes them suitable for H_2_ transport. The affinity of metals with H_2_ was not so obvious considering the lower permeance, and a further study will be required. 

The separation of alkane/alkene is a hot system and have drawn much attention by using Metal-Organic Frameworks (MOFs) membranes. BTESE membranes doped with metals have shown good separation performance with better hydrothermal ability than most MOFs. Al-doped bis(triethoxysilyl)methane (BTESM)-derived hybrid organosilica membranes were designed to separate C_3_H_6_/C_3_H_8_ via the incorporation of Al and using Si–C–Si units as a spacer method to precisely control silica network size. High C_3_H_6_/C_3_H_8_ permeance ratios of approximately 40 for Al-doped BTESM (Si/Al = 9/1) membranes were achieved. Ag^+^ doped into BTESM membranes was reported, and 10% Ag/BTESM showed the highest C_3_H_6_/C_3_H_8_ selectivity of 32.5 in a binary gas system [27,95]. 

The research of metal-doped hybrid organosilica membranes on the liquid-phase separation is rare, although great potential performance is expected. For example, 1–35 mol% Co-doped into BTESE membranes exhibited the ammonia rejection that was above 99% at 45 °C [96]. La_25_Y_75_-doped BTESE membranes showed very high water flux of 10.3 kg m^−2^ h^−1^ for pervaporation of 3.5 wt% NaCl solutions at 25 °C, while achieving rejections of almost 100% [85].

Metals can be easily doped into organosilica membranes via the sol-gel method, and have great potential in the improvement of separation performance. Although the effects that metal content and calcination temperature can exert on the pore size and affinity have been discussed with respect to the separation ability, factors including different types of metals and other synthesis conditions are needed to be further investigated to well elucidate the mechanisms of metal doping. 

### 3.2. Alkoxysilane Co-Condensation

TEOS and BTESE have received the most extensive amount of study among precursors for membrane preparation. To further improve the hydrothermal stability and performance, other alkoxysilanes containing –CH_3_, –OH or amino groups have been incorporated into TEOS and BTESE networks to modify the membranes. The main hybrid membranes and their applications are summarized in Table 6, and are discussed in a later section. 

Methyltriethoxysilane (MTES) with terminal methyl groups (Si–CH_3_) is the most studied material that is incorporated into TEOS or BTESE sols to prepare hybrid organosilica membranes [14,70,97,98,99,100]. These membranes exhibited great improvement in hydrothermal stability through –CH_3_ induced from MTES, which draw an attractive attention in practical gas and liquid applications in the presence of water molecules. The hydrophobic property and structure of micropores larger than those of pure silica membranes was obtained by adding MTES to TEOS sols (molar ratio: TEOS/MTES = 1) using an acid catalyst [97]. The formed Me-SiO_2_ membranes were preferred for the separation of H_2_ from larger gases. To further enhance the hydrothermal stability and H_2_ selectivity, metals such as Pd or Co together with MTES were incorporated into TEOS sols [98,100]. The Pd–Me–SiO_2_ membrane showed a super high H_2_ permeance of 1 × 10^−^^5^ mol m^−^^2^ s^−^^1^ Pa^−^^1^ with a media H_2_/N_2_ permselectivity of 11 at 200 °C. The performance was almost unchanged after steam exposure [100]. In the dehydration of 95 wt% butanol/water mixtures at 95 °C, the hydrothermal stability was also enhanced by incorporation of methyl groups in TEOS-derived silica membranes that the performance was kept stable for more than 18 months [101]. From above it can be observed that by the hydrolysis and copolymerization of TEOS and MTES using an acid catalyst forms a microporous membrane with high hydrothermal stability and excellent performance in gas and liquid-phase separation. 

**Figure 9:**
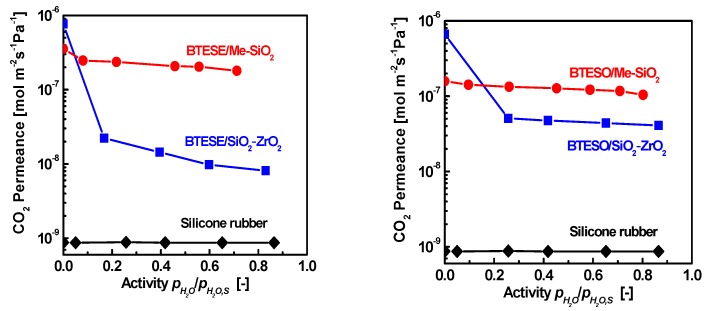
CO_2_ permeance of organosilica membranes as a function of water vapor activity at 40 °C (Feed side: 500 mL/min, permeate side: 1000 mL/min). Reproduced from [104].

When a base is used as the catalyst, a mesoporous network in the range of 1–5 nm is usually formed via sol-gel method [102]. Methylated silica membranes were prepared by mixing of TEOS and MTES (1:1) and using ammonia catalyst. These membranes showed pore sizes in the range of 1.7–4.3 nm, and a water contact angle as high as 120° [99]. In the nanofiltration of hexane from polyolefin oligomers, the permeability of hexane reached to 2.0–7.58×10^−11^ m^3^ m^−^^2^ s^−^^1^ Pa^−^^1^. In addition, Me–SiO_2_ with a nanometer pore size prepared by TEOS and MTMS (methyltrimethoxysilane) was used as hydrophobic intermediate layers instead of hydrophilic intermediate layer such as SiO_2_–ZrO_2_ to prepare BTESE and BTESO membranes for gas separation in humid process, as shown in Figure 9 [103,104,105]. The CO_2_ gas permeance was around 10^−7^ mol m^−2^ s^−1^ Pa^−1^ for both BTESE(O)/Me–SiO_2_ and BTESE(O)/SiO_2_–ZrO_2_ membranes in dry case. However, in the presence of water vapor (partial pressure: 0.1–0.8), the CO_2_ permeance was decreased to the order of 10^−^^9^–10^−^^8^ mol m^−^^2^ s^−^^1^ Pa^−^^1^ for BTESE(O)/SiO_2_-ZrO_2_ membranes, while the permeance was just little decreased and still around 10^-7^ mol m^−^^2^ s^−^^1^ Pa^−^^1^ for BTESE(O)/Me–SiO_2_ membranes with hydrophobic intermediate layer of Me–SiO_2_.

MTES incorporated into BTESE sols has been also investigated in the preparation of a hybrid organosilica membrane with molecular sieving property and hydrothermal stability [29,70,106,107]. For dehydration of alcohol/water (95/5 wt%) mixtures by pervaporation, the membranes showed initial water flux as high as 10 kg m^−^^2^ h^−^^1^ with permeated water concentration of 98 wt% for almost 2 years at 150 °C [106]. The hydrothermal stability was greatly improved. The pore size of these hybrid organosilica membranes can be tuned by the different ratio of BTESE and MTES. The highest selectivity of H_2_/N_2_ and H_2_/CH_4_ were found to be 64.4 and 73.6 when the molar ratio of BTESE: MTES was equal to 1:2 [29]. 

Moreover, HMTES with –OH groups was incorporated into BTESE to increase the hydrophilicity in desalination [32]. HMTES/BTESE membrane with a weight ratio of 1:1 showed a higher liquid permeance of 3.4 × 10^−13^ m^3^ m^−^^2^ s^−^^1^ Pa^−1^ and maintained NaCl rejection at 95.5%, compared with pure BTESE membranes with a permeance of 3.1 × 10^−14^ m^3^ m^−2^ s^−1^ Pa^−1^ and NaCl rejection at 95.2%. By incorporation of HMTES into more hydrophilic BTESEthy (Si–CH=CH–Si) with a double bond, new membranes of HMTES/BTESEthy (1:1) showed about 4 times higher liquid permeance (1.4 × 10^−12^ m^3^ m^−2^ s^−1^ Pa^−1^) than HMTES/BTESE membranes with a little lower NaCl rejection (89%) [108]. A novel layered-hybrid membrane that was created by depositing organosilica layers onto a flexible polymeric nanofiltration membrane as another hybrid configuration obtained a much higher water permeance of 1.2 × 10^−12^ m^3^ m^−2^ s^−1^ Pa^−1^ with NaCl rejection of 96% than that of HMTES/BTESE membranes [30]. 

**Figure 10:**
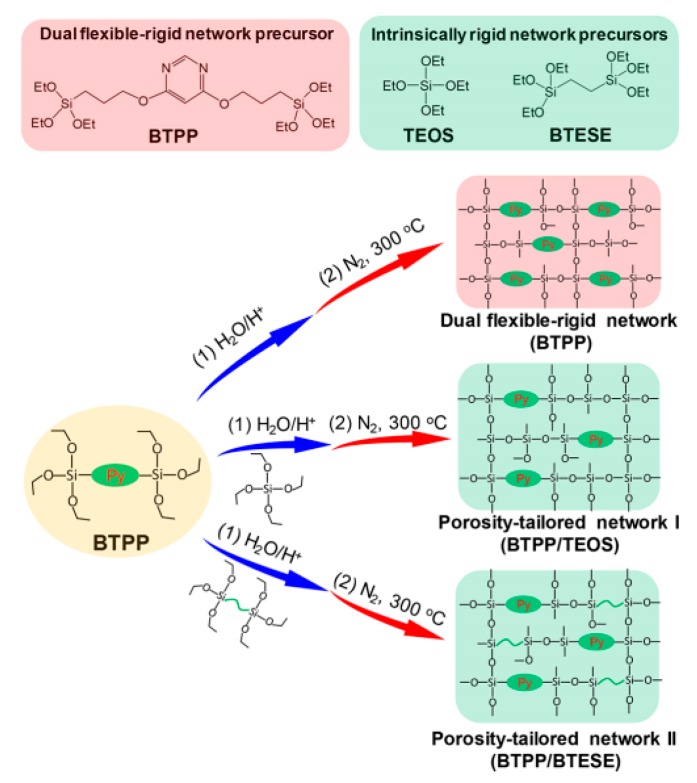
A porosity-tailored network via the co-condensation of a dual flexible−rigid network of BTPP and rigid network of TEOS and BTESE. Reproduced from [109].

Amine-containing materials have been investigated for their ability to enhance CO_2_ adsorption and water affinity. Tsuru’s group reported a dual flexible−rigid network of 4,6-bis(3-triethoxysilyl-1-propoxy)-1,3-pyrimidine (BTPP) containing steric amine groups incorporated into TEOS and BTESE membranes [109]. The network was formed via sol-gel method, as shown in Figure 10. These composite BTPP/TEOS and BTPP/BTESE membranes achieved a high CO_2_ permeance that was over 2000 GPU with a moderate CO_2_/N_2_ selectivity of 20. By adding pendant amine groups into organosilica membranes, the water affinity was also improved. For the pervaporation of 95 wt% BuOH/H_2_O, the hybrids BTESE/PA (3-aminopropyltriethoxysilane), BTESE/IM (N-(3-triethoxysilylpropyl)-4,5-dihydroimidazole) and BTESE/LDA (3-(2-aminoethyl-amino)propyl trimethoxysilane) membranes with amino groups showed water fluxes in the range of 1.6 to 6.2 kg m^−2^ h^−1^ and the permeated water concentrations were over 92 wt%, which were higher than that of pure BTESE membranes with water flux of 1.5 kg m^−2^ h^−1^ and permeated water concentration of 90 wt% [74].

In addition to the hydrothermal stability and hydrophilic property that can be optimized by the hybrid method, the pore size of membranes also can be tuned for desired separation. TEOS was incorporated into BTESE membrane in an attempt to separate Ag/Au nanoparticles [110] or to separate O_2_ from O_2_/SO_2_ mixtures [111]. An average membrane pore size for TEOS/BTESE (0.43 nm) was between BTESE (0.51 nm) and TEOS (0.34 nm) membranes based on NKP method evaluation. The TEOS/BTESE hybrid membranes exhibited O_2_ permeance of 1.72–2.87 × 10^−8^ mol m^−2^ s^−1^ Pa^−1^ with an O_2_/SO_2_ selectivity of 7.3 under testing temperature in the range of 150 to 300 °C.

### 3.3. Other Types of Hybrid Organosilica 

Inorganic ions and other functional materials incorporated into organosilica membranes have been reported, including F^-^, Carbon, MOF and POSS [112,113]. Fluorine-doped BTESM membranes affected the physiochemical properties, which became more hydrophobic with an enlarged network size compared with pure BTESM organosilica membranes without doping, which is suitable for separation of large molecules such as hydrocarbon gases [28]. The utilization of triethoxyfluorosilane (TREOFS) and TEOS mixtures to prepare F-SiO_2_ (F/Si = 1/9) membranes, which showed a CO_2_ permeance of 4.1 × 10^−7^ mol m^−2^ s^−1^ Pa^−1^ with high CO_2_/CH_4_ selectivity of 300 at 35 °C [114]. The membrane BTESE/POSS prepared by hydrolysis/condensation of POSS and BTESE were robust to heat and chlorine. Their performance was unchanged even after the membrane exposure to 10,000 ppm·h aqueous NaOCl [115]. Recently, hydrophobic polydimethylsiloxane (PDMS) and hydrophilic poly(ethylene glycol)(PEG) cross-linked with BTESE have been studied. The hydrophobic PDMS/BTESE hybrid membrane exhibited superior durability in desulfurization by pervaporation of thiophene/n-octane mixtures (gasoline model) [116]. The PEG/BTESE membranes became more hydrophilic, from water contact angle of 60° for pure BTESE to 25° for PEG/BTESE by 20% PEG adding, which resulted in a high level of water permeability in desalination by reverse osmosis [117].

By doping with functional materials, these hybrid organosilica membranes exhibited different properties, which enhanced their performance and expanded the scope of their applications. Till now, the types of hybrid materials remain limited. Predicting the properties of hybrid membranes and understanding of transport mechanisms should be further explored. 

## 4. Conclusions and Prospects 

Organosilica and hybrid organosilica membranes and their use in gas and liquid-phase separation are focused in this review. The organosilica membranes with organic groups present excellent hydrothermal stability in steam and liquid at high temperature. Optimizing the pore sizes and chemical properties of organosilica membranes by doping with metals, alkoxysilanes or functional materials are an efficient and simple method to improve the gas and liquid separation performance for desired applications. Novel one- and two-dimension materials, such as graphene oxide, carbon nanotubes, and silica nanosheets which may provide more properties for organosilica membranes will be interesting to investigate in the future for potential applications.

## Figures and Tables

**Table 1:** Effect of the parameters in the sol-gel process.

Composition	Water Ratio, Acid Ratio, Solvent Ratio
Calcination temperature	100–600 °C
Other method factors	Spin-coating, UV-irradiation

**Table 2:** Bridged-organosilica membranes and their performance in gas-phase separation.

Precursor	Organic Group	T [°C]	H_2_ Permeance [10^−7^ mol m^−2^ s^−1^ Pa^−1^]	Selectivity	Ref.
BTESM	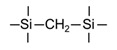	200	~10	H_2_/N_2_: 70	[15]
				H_2_/CH_4_: 150	
		200 50	2.4 6.32 ^a^ 17.9	H_2_/N_2_: 20.7 C_3_H_6_/C_3_H_8_: 8.8 H_2_/N_2_: 12	[61] [62] [63]
BTESE	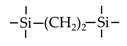	200 200 40 200 200 200	11 ~10 7.66 20–100 2.2 16–34	H_2_/CH_4_: 150 CO_2_/N_2_: 36 H_2_/CH_4_: >400 H_2_/N_2_: ~20 H_2_/SF_6_: 1000–25500 CO_2_/N_2_: >100 H_2_/C_3_H_8_: 2600–5800	[61] [15] [16] [26] [37] [39]
BTESP	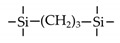	200	~8	H_2_/CH_4_: ~50	[15]
BTMSH	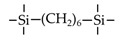	200	~2.5	H_2_/CH_4_: ~10	[15]
BTESB	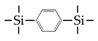	200 200	2 15.2	H_2_/CH_4_: ~25 H_2_/N_2_: 8.4	[15] [63]
BTESO	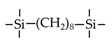	200 40	~7 6.63 23.2	H_2_/CH_4_: ~5 CO_2_/N_2_: ~12 H_2_/N_2_: 5.3	[15] [16] [17]
BTESEthy	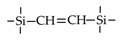	200	17	H_2_/N_2_: 40	[64]
BTSEA	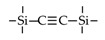	200	26.8	H_2_/N_2_: 10.3	[19]
BTMSN	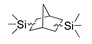	200	7	H_2_/N_2_: 12	[65]
BTES-ED	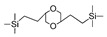	200	6	H_2_/N_2_: 11 H_2_/SF_6_: 1160	[65]
BTES-MAz	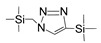	50	0.0295	C_3_H_6_/C_3_H_8_: 37	[66]
BTPP	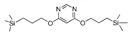	35	~0.6	H_2_/N_2_: ~50CO_2_/N_2_: 25	[67]

Ref.: reference; ^a^: C_3_H_6_. The other groups Si connected is –OCH_2_CH_3_ or –OCH_3_, and the whole name is put in the Abbreviations.

**Table 3:** Bridged-organosilica membranes and their performance in liquid-phase separation.

Precursor	Organic Group	Separation System	T [°C]	Flux [kg m^−2^ h^−1^] /Perm [10^−13^ m^3^ m^−2^ s^−1^ Pa^−1^]	Permeated H_2_O%/Rejection	Ref.
BTESM	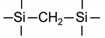	95%EtOH/H_2_O ^a^	70	1.18	92.2%	[61]
BTESE	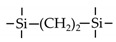	95%EtOH/H_2_O ^a^ 90%IPA/H_2_O ^a^ 90%IPA/H_2_O ^a^ 95%BuOH/H_2_O ^a^ 2% NaCl ^b^	70 75 75 70 80	0.73 3.54 1.9–3 20 10	89.2% 96% 96–99% 99% 98.1%	[61] [68] [69] [70] [71]
BTESB	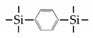	95%BuOH/H_2_O ^a^	95	3	32.6	[17]
BTESO	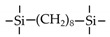	95%BuOH/H_2_O ^a^	95	3.2	92.7	[17]
BTESEthy	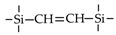	2%NaCl ^a^ 2% NaCl ^b^	70 25	14.2 2	99.6% 97%	[64] [19]
BTSEA	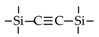	2% NaCl ^b^	25	8.5	95%	[19]
BTMSN	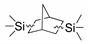	2%NaCl ^b^	25	0.1	95%	[72]
BTES-ED	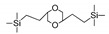	2%NaCl ^b^	25	1.84	98.5%	[65]
BTES-MAz TTESPT	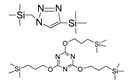	2%NaCl ^b^ 2%NaCl ^b^	25 60	3.7–5.4 >10	95–96% >98.5	[73] [63]

EtOH: ethanol; IPA: isopropanol; Bu: butanol; Perm: permeability; Ref.: reference. ^a^: Pervaporation, Flux (fifth column), Permeated H_2_O% (sixth column); ^b^: Reverse osmosis; Perm (fifth column), Rejection (sixth column). The other groups Si connected is –OCH_2_CH_3_ or –OCH_3_, and the whole name is put in the Abbreviations.

**Table 4:** Pendant-organosilica membranes and their performance in gas-phase separation.

Precursor	Organic Group	T [°C]	Permeance [10^−7^ mol m^−2^ s^−1^ Pa^−1^]	Selectivity	Ref.
MTES	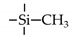	200	H_2_: 5	H_2_/N_2_: 15 H_2_/C_3_H_8_: 1300	[51]
PhTES	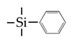	200	H_2_: 2	H_2_/N_2_: 7.4 H_2_/C_3_H_8_: 13.1	[51]
MTMS	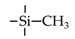	25	H_2_: 0.09	He/N_2_: 15 *	[58]
DMDMS	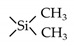	500	H_2_: 2.8	H_2_/N_2_: 2000 *	[49]
TMMS	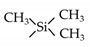	25	H_2_: 0.1	He/N_2_: 7.8 *	[58]
APTES	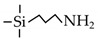	300	H_2_: ~1.5	H_2_/N_2_: 13.9	[74]
SA	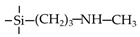	35	CO_2_: 0.17	CO_2_/N_2_: 11	[22]
TA	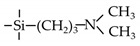	35	CO_2_: 1.72	CO_2_/N_2_: 21	[22]
QA	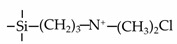	35	CO_2_: 0.52	CO_2_/N_2_: 24	[22]
TEFS	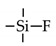	35	C_3_H_6_: 2.2	C_3_H_6_/C_3_H_8_: 42	[21]

*: CVD; Ref.: reference. The other groups Si connected is –OCH_2_CH_3_ or –OCH_3_, and the whole name is put in the Abbreviations.

**Table 5:** Pendant-organosilica membranes and their performance in liquid-phase separation.

Precursor	Organic Group	Separation System	T [°C]	Flux [ kg m^−2^ h^−1^] /Perm [10^−13^ m^3^ m^−2^ s^−1^ Pa^−1^]	Permeated H_2_O%/Rejection	Ref.
APTES	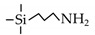	95%EtOH/H_2_O ^a^	70	0.2–2.1	~90%	[74]
HMTES	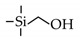	2%NaCl ^b^	25	7.3	86.4%	[32]
IM	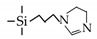	95%EtOH/H_2_O ^a^	70	1–3.4	~95%	[74]
LDA	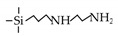	95%EtOH/H_2_O ^a^	70	2.2–4.1	~94%	[74]

EtOH: ethanol; Perm: permeability; Ref.: reference. ^a^: Pervaporation, Flux (fifth column), Permeated H_2_O% (sixth column); ^b^: Reverse osmosis; Perm (fifth column), Rejection (sixth column). The other groups Si connected is –OCH_2_CH_3_ or –OCH_3_, and the whole name is put in the Abbreviations.

**Table 6:** The main reported hybrid membranes and their applications.

Hybrid Material	Application	Separation System
MTES/TEOS	Gas separation; PV Nanofiltration	H_2_/other gas; Dehydration of alcohol/water Hexane/polyolefin oligomers
MTMS/TEOS	Intermediate layer	CO_2_/N_2_ in water vapor
BTESE/TEOS	Gas separation	O_2_/SO_2_
BTPP/TEOS	Gas separation	CO_2_/other gas
BTPP/BTESE	Gas separation	CO_2_/other gas
MTES/BTESE	PV Gas separation	Dehydration of alcohol/water H_2_/other gas
HMTES/BTESE	RO	H_2_O/NaCl
HMTES/BTESEthy	RO	H_2_O/NaCl

## Abbreviations

APTES or PA	3-aminopropyltriethoxysilane
BTESM	bis(triethoxysilyl)methane
BTESE	1,2-bis(triethoxysilyl)ethane
BTESP	1,3-bis(triethoxysilyl)propane
BTMSH	1,6-bis(trimethoxysilyl)hexane
BTESB	bis(triethoxysilyl)benzene
BTESO	1,8-bis(triethoxysilyl)octane
BTESEthy	1,2-bis(triethoxysilyl)ethylene
BTESA	1,2-bis(triethoxysilyl)acetylene
BTES-ED	2,5-bis[2 -(triethoxysilyl)ethyl]-1,4-dioxane
BTES-MAz	1,4-bis(triethoxysilylmethyl)-1,2,3-triazole
BTMES	bis(trimethoxysilyl)ethane
BTMSN or BTMS-Nor	bis(trimethoxysilyl)norbornane
BTPP	4,6-bis(3-triethoxysilyl-1-propoxy)-1,3-pyrimidine
DMDMS	dimethoxydimethylsilane
DMDPS	dimethoxydiphenylsilane
HMDS or HMDSO	Hexamethyldisiloxane
HMTES	hydroxymethyl(triethoxy)silane
IM	N-(3-triethoxysilylpropyl)-4,5-dihydroimidazole
LDA	3-(2-aminoethylamino)propyl-trimethoxysilane
MTES	methyltriethoxysilane
MTMS, or MTMOS	methyltrimethoxysilane
PhTES	phenyltriethoxysilane
PTMS	phenyltrimethoxysilane
QA	3-(triethoxysilyl)-N, N-dimethylpropan-1-amine
SA	4,6-bis(3-triethoxysilyl-1-propoxy)-1,3-pyrimidine
TA	3-(triethoxysilyl)-N-methylpropan-1-amine
TEFS	triethoxyfluorosilane
TMMS, or TMMOS	trimethylmethoxysilane
TRIES	triethoxysilane
TTESPT	2,4,6-tris[3-(triethoxysilyl)-1-propoxy]-1,3,5-triazine
TPMS	triphenylmethoxysilane
